# Impact of NRTI backbone on renal, bone and cardiovascular markers in HIV-infected individuals receiving a boosted protease inhibitor

**DOI:** 10.7448/IAS.17.4.19562

**Published:** 2014-11-02

**Authors:** Tristan Barber, Andrew Hill, Gurmit Jagjit Singh, Marta Boffito, Mark Nelson, Graeme Moyle

**Affiliations:** Chelsea and Westminster NHS Foundation Trust, St Stephen's AIDS Trust Clinical Trials Unit, London, UK

## Abstract

**Introduction:**

We have previously shown in the SSAT 044 study that unconjugated hyperbilirubinaemia in subjects receiving a boosted protease inhibitor (PI/r) has limited impact on renal, cardiovascular (CV) and bone biomarkers, as well as on neurocognitive performance, relative to those receiving PI/r with a normal bilirubin. We present here a secondary analysis comparing markers in those receiving abacavir- vs tenofovir- based antiretroviral therapy (ART).

**Materials and Methods:**

This cross-sectional study included 101 HIV-1 infected individuals stable (HIV RNA<50 cps/ml, >6 months) on antiretroviral regimens including tenofovir (TDF)/emtricitabine or abacavir/lamivudine plus a ritonavir boosted PI.

**Results:**

Forty-three subjects had normal bilirubin (NBR) levels and 35 had high bilirubin (>2.5 times upper limit); the remaining 23 patients had intermediate bilirubin levels or violated the protocol. The mean age of participants was 48 years; 93% were male and 84% Caucasian; 22 received ABC-based therapy and 78 TDF. No differences were seen in cardiovascular markers: Framingham (10-year risk % median, IQR): ABC 8.1, 5.6–15.3; TDF 9.5, 4.8–13.4 (p=ns); pulse wave velocity and carotid intimal thickness also showed no significant differences. No differences were seen in bone parameters: Calcaneal Stiffness Index (median score, IQR): ABC −0.5, −0.8 to 0.8; TDF −0.5, 1.4–0.4 (p=ns); 10 year FRAX score (% median, IQR): ABC 5.0, 2.4–6.2; TDF 3.6, 2.5–5.8 (p=ns). There were differences in renal parameters as shown in Table 1. We show statistically significant differences in urine protein/creatinine ratio (uPCR) (10 vs 7; p=0.004) and urine albumin/creatinine ratio (uACR) (15 vs 8; p=0.002), with both being higher in the TDF group.

**Conclusions:**

Tenofovir use is associated with excess loss of proteins including those typically resorbed in the renal tubule. Abacavir use was not associated with an increase in biomarkers of CV risk or vascular dysfunction.

**Table 1:** Differences in renal parameters

	ABC use		No ABC use		
					
Parameter	Median (IQR)	N	Median (IQR)	N	p Value
Protein/creatinine ratio (mg/mmol)	7 (6–9)	18	10 (8–14)	67	0.004
Urinary retinol binding protein/creatinine ratio (ug/mmol)	8 (6–10)	19	15 (8–240)	67	0.002
	ABC use		No ABC use		
Parameter	Number normal (%)	N	Number normal (%)	N	p Value
Protein/creatinine ratio (mg/mmol)	16 (89)	18	49 (73)	67	0.137
Urinary retinol binding protein/creatinine ratio (ug/mmol)	19 (100)	19	50 (75)	67	0.008

